# Yawning and Penile Erection Frequencies Are Resilient to Maternal Care Manipulation in the High-Yawning Subline of Sprague–Dawley Rats

**DOI:** 10.3389/fnbeh.2020.00020

**Published:** 2020-03-12

**Authors:** Ángeles Dorantes-Nieto, Carmen Cortes, Araceli Ugarte, Angélica Trujillo Hernández, Ángeles Carrasco, Héctor Alejandro Cepeda-Freyre, Jose R. Eguibar

**Affiliations:** ^1^Institute of Physiology, Benemérita Universidad Autónoma de Puebla, Puebla, Mexico; ^2^Biological Sciences Faculty, Benemérita Universidad Autónoma de Puebla, Puebla, Mexico; ^3^Research Office, Vice-rectory of Research and Postgraduate Studies, Benemérita Universidad Autónoma de Puebla, Puebla, Mexico

**Keywords:** anxiety, sexual behavior, maternal care, grooming, epigenetic, cross-fostering, depression, scratching

## Abstract

Yawning is a stereotyped behavioral pattern characterized by wide opening of the mouth associated with deep inspiration followed by short expiration. All vertebrate species yawn, but with low frequencies. We obtained two sublines of Sprague–Dawley (SD) rats by a strict inbreeding process: one with a high-yawning frequency (HY) of 20 yawns/h, which is one order of magnitude higher with respect to the low-yawning frequency (LY) subline, with 2 yawns/h. Outbred SD rats had a yawning frequency of 1 yawn/h. HY dams had a different organization of maternal care with respect to that displayed by LY and SD dams because HY dams constructed lower quality nests and had more re-retrieving and atypical retrieving. The aim of this study was to analyze the changes in maternal care using in- and cross-fostering between the sublines and SD dams and to measure spontaneous and dopaminergic-induced yawning, penile erections, grooming and scratching bouts. We also measured the expression of dopamine D_2_ receptors in the striatum using Western blot analysis. Our results showed that HY male rats reared by SD or LY dams did not significantly differ in yawning frequencies with respect to HY male rats reared by mothers of their own phenotype. Maternal care did not differ between sublines and SD dams independent of the litter they reared. However, LY rats reared by HY dams showed a significant increase in the number of spontaneous penile erections. Importantly, in-fostered HY male rats had the highest number of yawns induced by systemic administration of (−)-quinpirole supporting that higher maternal care display can influence the frequency of dopaminergic-induced yawning. In fact HY male rats in all conditions yawned more than did LY and SD male rats independent of the dam that raised them supporting a strong influence of genetic background. However SD male rats raised by LY dams showed significantly increased the dopamine D_2_ receptor expression. In conclusion, maternal care and the environmental nest conditions during the lactation period did not change the phenotypic characteristics of the yawning sublines supporting that their genetic background is fundamental for the expression of spontaneous or dopaminergic-induced yawning.

## Introduction

Yawning is a stereotyped behavioral pattern that is characterized by deep inspiration followed by short expiration across all vertebrate species (Barbizet, 1958; Argiolas and Melis, 1998; Collins and Eguibar, 2010). In mammals, including rats, yawning frequency is very low, approximately 1 yawn/h (Baenninger, 1997).

We selectively inbred for more than 85 generations two sublines of Sprague–Dawley (SD) rats: a high-yawning (HY) subline, with a mean of 20 yawns/h, which is one order of magnitude higher than the low-yawning (LY) subline, with a mean of 2 yawns/h (Urbá-Holmgren et al., 1990; Eguibar et al., 2015). We used as a control group and outbred SD rats that have a mean of 1 yawn/h. HY rats allow us to analyze the environmental influences on yawning behavior. Yawning frequency has a circadian rhythm with a peak before dusk (Anías et al., 1984). The yawning circadian rhythm is not an endogenous mechanism because it is not free running, and it can be synchronized by a restricted feeding period, with food availability being a stronger zeitgeber than a light–dark cycle (Holmgren et al., 1991). These sublines also differ in their responses to stress, as seen in the open-field arena, where HY rats are more active than LY rats, indicating that HY rats are less emotionally reactive than the latter (Moyaho et al., 1995).

The physiological role of yawning has not been clearly established until now, and there are several hypotheses about the role of this innate motor pattern in respiratory and circulatory roles. Several studies have demonstrated that yawning can change the respiratory rhythm, increasing heart frequency and blood oxygenation, as well as vasodilatation, but these variables are not triggering yawning. In conclusion, there is not a clear physiological association between respiration or circulation and yawning (Guggisberg et al., 2010). Another hypothesis about the physiological role of yawning is the role of drowsiness as an inducer of yawning and a concomitant increase in arousal levels as a method of global activation of brain activity from brain stem to cortical areas, but there is no convincing evidence of such an association (Guggisberg et al., 2010). A third hypothesis is the role of yawning in cooling down the brain temperature, the so-called thermoregulatory hypothesis (Gallup and Gallup, 2008). Based on the high yawning frequency of HY male rats, we can demonstrate that when a yawn happens, there is a decrease in the cornea and ear concha temperatures, two hairless facial structures, using thermographic analysis. In fact, 10 s after a yawn happens, there is a reduction in both facial areas that correlates with the reduction in the cortical temperature measured with implanted thermocouple probes in non-HY rats that then returns to basal levels within a short time period (Shoup-Knox et al., 2010; Eguibar et al., 2017b). In another study, we demonstrated in budgerigars that the beak temperature decreased when a yawn happened (Gallup et al., 2017). These experimental data indirectly support the thermoregulatory role of yawning, but it is necessary to obtain more empirical data to support the hypothesis. Finally, the blood gas (CO_2_/O_2_) hypothesis was rejected because breathing neither pure oxygen nor high CO_2_ had a significant effect on yawning frequency, although both increased the breathing rate (Provine et al., 1987).

On the other hand, HY male rats allow us to show that this innate behavioral pattern is strongly correlated with spontaneous penile erections in a very short time window of only 3 min; in fact, 50% of yawns and penile erections happen together (Holmgren et al., 1985). Yawning frequency also correlated with the number of penile erections after systemic administration of D_2_-like dopaminergic agonists such as apomorphine in low doses, bromocriptine, or (−)-quinpirole (Urbá-Holmgren et al., 1993; Eguibar et al., 2003); this behavioral correlation is present even in a myelin mutant rat with progressive demyelination (Eguibar et al., 2012).

Yawning behavior is regulated by several neurotransmitters, including cholinergic, muscarinic, or D_2_-like dopaminergic agonists; it is inhibited by opioids and GABAergic mechanisms (for review, see Argiolas and Melis, 1998; Collins and Eguibar, 2010); and it is increased by the central administration of adrenocorticotrophic, α-melanocyte-stimulating hormone (MSH), oxytocin, and prolactin peptides and inhibited by bombesin (Argiolas and Melis, 1998; Díaz-Romero et al., 2002; Collins and Eguibar, 2010). Among all of these neurotransmitters and neuromodulators, the dopaminergic system, through D_2_-like receptors, is the most potent inducer of yawning and penile erections, acting in the paraventricular nucleus (PVN) of the hypothalamus (Sanna et al., 2012), and the motor output is regulated by the striatum (Dourish and Cooper, 1990).

On the other hand, yawning is part of a behavioral syndrome induced by exposure to a stressor, with a strict temporal organization and an initial increase in alertness, grooming, yawning, and finally somnolence or even sleep (Delius, 1988). Therefore, grooming and yawning are two behaviors that reduce stress responses and have adaptive properties (Fentress, 1988). In the case of HY rats, they groomed more when exposed to a novel environment (Eguibar and Moyaho, 1997) or after wetting the fur (Moyaho et al., 1995), indicating that they had different coping strategies to confront a stressor. This approach is supported when HY rats are exposed to an open-field arena because they ambulate more and have a lower number of fecal boluses; therefore, they are less emotionally reactive (Moyaho et al., 1995). This finding is corroborated by a preliminary study in which HY rats explore more the open arms in the elevated-plus maze, supporting that they are less anxious (Eguibar et al., 2017a).

It is well established that maternal care plays a substantial role in physiological and behavioral stress responses in adulthood (Caldji et al., 2000). In this context, HY dams spent less time in the nest, retrieved their pups faster, and showed a longer latency to licking and mouthing the pups than LY dams. The HY dams also had atypical retrieving, and they built nests of less quality, supporting that they are motivated to take care of their pups, but the “fine tuning” of maternal care is different (Ugarte et al., 2011). In a previous study, we analyzed yawning frequencies in male and female rats after cross-fostering, but all subjects were raised in individual cages to increase their stress responses. Our results showed that sex ratio and littermate size influenced yawning frequency in adulthood (Moyaho et al., 2009), but no further analysis was performed until now.

Based on these data, the aim of this study was to first analyze the role of maternal care in spontaneous yawning and penile erections in male rats raised in conditions of in- and cross-fostering between the sublines or with SD dams. Second, we analyzed the maternal behavior during the initial phase of the lactation period in conditions of in- or cross- fostering between the sublines or with SD dams. Third, in another group of rats, fostered by different types of dams, we determined yawning, penile erection, grooming, and scratching frequencies after systemic administration of a specific D_2_-like receptor agonist (−)-quinpirole, and finally in another group of rats, we measured the relative expression of D_2_ receptors in the striatum using Western blot analysis.

## Materials and Methods

### Animals

We used a total of 198 HY, LY, and SD male rats, with 63 being dams. For the (−)-quinpirole experiments, we evaluated 99 rats: 33 HY, 33 LY, and 33 SD. Additionally, we used 36 rats for Western blot experiments: 12 HY, 12 LY, and 12 SD. All subjects (Ss) were bred in our animal room facilities at the Institute of Physiology of the Benemérita Universidad Autonóma de Puebla. After weaning (28 days), the rats were maintained at 3–4 rats per transparent acrylic cage (46 × 32 × 20 cm), with the floor covered with wood shavings (Beta Chip, Warrensburg, NY, USA). The Ss were maintained in a room with controlled temperature (22°C ± 2°C) and relative humidity between 30% and 45%. Ss were maintained under a 12:12 h light–dark schedule (lights on at 07:00), with free access to balanced rodent pellets (Purina Mills 5001, Richmond, IN, USA) and purified water (Ciel, FEMSA, Estado de México, México), and they weighed between 280 and 310 g when the experiment began. All trials were conducted in the light phase between 10:00 and 13:00.

All procedures described have been performed in compliance with the Laws and Codes approved in the seventh title of the Regulations of the General Law of Health regarding Health Research of the Mexican Government (NOM-062-ZOO-1999) and following the National Institutes of Health Guide for the Care and Use of Laboratory Animals (eighth edition, 2011). All experiments procedures were approved by Benemérita Universidad Autónoma de Puebla Animal Care and Use Committee, no. EGC SAL-G-2019.

### In- or Cross-fostering Procedures and Measuring Maternal Behavior

**Figure 1:**
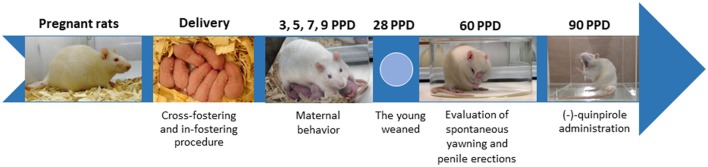
Experimental design. Pregnant female rats from the high-yawning (HY), low-yawning (LY), and Sprague–Dawley (SD) lines were maintained in individual acrylic cages until they delivered the pups. In the first 8 h, the pups were culled to eight pups (four males and four females), and they were weaned at 28 days of age. At 60 days of age, we evaluated spontaneous yawning and penile erections in a glass cylinder, and later, the dose-response to systemic (−)-quinpirole administration was evaluated in a novel environment. PPD, post-partum days.

Pregnant dams were housed individually in transparent acrylic cages (46 × 32 × 20 cm; Figure 1). As soon as parturition happened, the pups were weighed and sexed, and the litter was curled to four females and four males and swapped between cross- and in-fostered dams. Therefore, HY, LY, or SD litters were fostered to SD dam mothers of the same subline or SD (in-fostered) or to mothers of the opposite subline or SD (cross-fostered). Then, the pups were returned to the dams until weaning at 28 days of age and housed four males/collective acrylic cage (46 × 32 × 20 cm). Spontaneous yawning and penile erections were determined at 60 days of age following the procedure described by Holmgren et al. (1985), and dopaminergic-induced yawning was determined at 90 days of age in a novel cage environment following previous procedures (see Experimental design in Figure 1; Eguibar et al., 2015).

For maternal behavior analysis, all dams were tested inside the maternal cage without disturbing them between 10:00 and 12:00 to minimize stress responses. Maternal behavior was observed for 20 min and videotaped using a Sony HD camera (model HDR-PJ260V, Tokyo, Japan) at postpartum days 3, 5, 7, and 9. All videos were stored on an internal HD of a PC computer under Windows 10 software for ulterior analysis. The behavioral analyses were performed using Observer XT software v. 11.0 (Noldus Information Technology, Wageningen, The Netherlands). All measurements were performed offline by a trainer observer who was blinded to dam characteristics and evaluated the percentage of time that the dam spent in different maternal behaviors, such as arched-back nursing, blanket-nursing, and side-nursing postures. We also evaluated licking and grooming of the young, self-grooming inside the nest, food intake, and time spent outside the nest area. In the video digital records, we measured the total amount of time spent in the following behaviors: nursing, body licking, genital licking, the time that the dams spent outside the nest, and nest building.

### Evaluation of Spontaneous Yawning and Penile Erections in Young Adult Male Rats

First, in all Ss, we determined their spontaneous yawning frequency at 2 months of age in a transparent glass cylinder (diameter 190 mm, height 100 mm) in which the floor was covered with a sheet of clean filter paper and the top was covered with a Plexiglas plate, leaving a 1-cm-wide gap for ventilation. The standard period of observation was 1 h, starting at 09:00, and the number of yawns, penile erections, and grooming and scratching bouts were recorded. Yawning was characterized as a prolonged (~1 s) wide opening of the mouth accompanied by deep inspiration and sometimes associated with tongue protrusion (Ushijima et al., 1984). The yawn ended when rats closed their mouths and returned to normal breathing (Holmgren et al., 1985; Eguibar et al., 2012, 2015). Penile erection consisted of pelvic thrusts immediately followed by the rat sitting in an upright position, an engorged penis, and subsequent licking of the perigenital area and even ingesting the ejaculate (Bagdy and Makara, 1995). A mirror was placed behind two stacked glass cylinders to allow the simultaneous observation of four rats by a trained observer following previous criteria (Eguibar et al., 2015; Figure 1).

### Dose-Response Analysis of Systemic (−)-Quinpirole, A Specific D_2_-Dopaminergic Agonist That Produces Yawning, Penile Erections, Grooming, and Scratching

One month after the spontaneous yawning frequencies evaluation, all Ss were distributed randomly from the different fostered groups. For the determination of the dose–response curve to the intraperitoneal (i.p.) injection of (−)-quinpirole hydrochloride (RBI, Inc., Natick, MA, USA; hereafter quinpirole), the drug was dissolved in sterile water with a constant volume of 1 mL/kg of body weight. We administered an increasing dose–response scheme of quinpirole hydrochloride administered every half hour in the order 0, 25, 50 and 100 μg/kg following the procedure used by two other groups and it is possible to use a cumulative dose scheme with (−)-quinpirole D_2_ agonist (Baladi and France, 2009; Serafine et al., 2015). In brief all observations started at 09:00 in an observation area that was 1 m from the housing place. The Ss were placed inside of a transparent acrylic cage 23 × 20 × 20 (cm), and the upper part of the cage was covered with a lid of the same material to allow ventilation. Under these circumstances, stress responses were reduced to a minimum. We injected i.p. each rat, and then behavior was continuously observed over a period lasting 30 min, and then the second i.p. dose was administered. We repeated the procedure until the four doses had been administered to the same animal, with a total experiment duration of 2 h 15 min. All sessions were also recorded with a Sony HD camera model HDR-PJ260 V, Tokyo, Japan and stored on the HD of a personal computer for analysis offline by a trained and blinded observer using Observer XT software v. 11.0; the number of yawns and penile erections, as well as the number of grooming and scratching bouts, were recorded.

A grooming episode was scored, as previously described (Eguibar et al., 2004), when any of the following components occurred: face washing, which consisted of vibrating movements of the forepaws in front of the snout, licking of the same paws followed by strokes along the snout, and semicircular movements over the top of the head; and body grooming, which consisted of the licking of body fur, genital grooming when licking the genital area, and paw licking of the forepaws and hindpaws. We also analyzed the scratching of the neck and thoracic body areas with the alternating pattern of hind limbs and licking the toes. Interruptions greater than 5 s determined separate grooming bouts.

### Relative Expression of the D_2_ Receptor in the Striatum of HY, LY, and SD Rats

We evaluated 12 rats from cross-fostered groups of male rats from each subline as well as SD rats. The rats were rapidly decapitated with a guillotine; the brains were obtained in cold conditions and kept on ice, and the striatum was dissected, placed in an Eppendorf tube, and stored at −80°C until measurements were performed. Total protein extracts were prepared from the striata by disruption in lysis buffer (20 mM Tris-HCl pH 7.4, 100 mM glycine, 100 mM NaCl, 0.1% Triton X-100, 1 mM phenylmethylsulfonyl fluoride, 1 mM DL-dithiothreitol), added with a protease inhibitor cocktail, with an electronic homogenizer (TissueTearor; BioSpec Products, Inc. IL, USA). Equal amounts of protein (70 μg by group) were denatured in Laemmli’s sample buffer, separated through 10% sodium dodecyl sulfate–polyacrylamide gels and electroblotted to nitrocellulose membranes (Bio-Rad Laboratories Headquarters, Mexico City, México). Blots were stained with Ponceau red (Amresco; 0.3% in acetic acid 1%) to confirm that the protein content was equal in all lines. Membranes were soaked in phosphate-buffered saline (PBS; containing 0.2% Tween-20) and incubated in 5% dry milk diluted in PBS for 1 h to block nonspecific protein-binding sites. Membranes were incubated overnight at 4°C with the primary antibody (rabbit polyclonal anti–rat dopamine receptor D_2_L, 1:200; cat no. AB1792P; Thermo Fisher Scientific Inc., Mexico City, México) diluted with milk 1% in PBS followed by secondary antibody (goat anti–rabbit immunoglobulin–horseradish peroxidase, 1:2,000; cat no. SC 2357; Industrias Bioselect, Mexico City) for 2 h. Immunoreactive polypeptides were detected using a chemiluminescence kit (West Pico Signal; Thermo Fisher Scientific, Monterrey, México) and were exposed to a Carestream Medical X-ray film. The relative expression of the D_2_ receptor was measured by densitometry and normalized against the signal obtained from Ponceau red staining used as loaded control (Romero-Calvo et al., 2010); for this, ImageJ software (National Institutes of Health, Bethesda, MD, USA) was used. Data are presented as the percentage of change against the control group. Measurements were done in two independent experiments and averaged, afterward.

### Statistical Analysis

All statistical analyses were performed using the R statistical environment (R Core Team, 2019). *P* ≤ 0.05 was accepted as indicative of a significant difference. All data are presented as the mean ± SEM unless otherwise stated.

### Experiment 1. Basal Spontaneous Yawning Frequency, Penile Erection and Grooming of In-fostered and Cross-fostered Male Rats Raised by Different Dams

For each of the behaviors, three different effects were tested independently using the Kruskal–Wallis test: the effect of pup type (HY, LY, or SD), the effect of dam subline (HY, LY, or SD), and the effect of the cross-in-fostering manipulation (manipulated or control). When a test detected a significant effect, *post hoc* group comparisons were performed using Dunn test with Holm–Šidák correction.

### Experiment 2. Maternal Care Organization in the In- or Cross-fostering Conditions Between the Sublines or With the SD Dams

Maternal components were modeled as proportions in the (0.1) interval by dividing the time each dam spent in each component over the total observation time. The proportions were then used as the outcome variable in hierarchical regression using a generalized linear model (GLM) of the beta distribution family. Dam subline, pup strain, and in- or cross-fostering manipulation were used as predictive factors.

### Experiment 3. Systemic Administration of Quinpirole Differentially Increased Yawning and Penile Erection and Decreased Grooming and Scratching Frequencies in Both Sublines and SD Rats

All statistical analyses were performed to test for two predictive factors: pup strain (i.e., HY, LY, or SD) and the strain of the foster mother (i.e., HY, LY, or SD). All behavioral data were analyzed using a GLM using Poisson, beta, or binomial families depending on the empirical distribution of the data (Quinn and Keough, 2003). The number of yawns, penile erections, and grooming or scratching bouts were each used as a dependent variable, and the strain of the foster mother or the type of male rats were the predictors. All second-order interactions were tested for inclusion in the models.

The effective dose (ED_50_) was defined as the estimated dose within the model expected to produce 50% of the maximum behavioral response measured in each of the dependent variables. The only exception was penile erections, which were modeled as a binary response, and the ED_50_ in this case represented the dose at which the probability of occurrence was estimated at 50%.

Some coefficients are presented as a change in log-odds, which represents the change in probability after undergoing a logit transformation, which consists of transforming probability into odds (event probability/1 − event probability) and then odds into log-odds (logarithm base 10 of the odds).

### Experiment 4. Dopamine D_2_ Receptor Expression in the Striatum

The levels of D_2_ receptor expression in the striatum were analyzed using Kruskal–Wallis analysis of variance (ANOVA) followed by Dunn multiple-comparisons test using Prism software v. 7 for Windows 10, San Diego, CA, USA.

## Results

### Experiment 1. Basal Spontaneous Yawning Frequency, Penile Erection, and the Grooming of In-fostered and Cross-fostered Male Rats Raised by Different Dams

**Figure 2:**
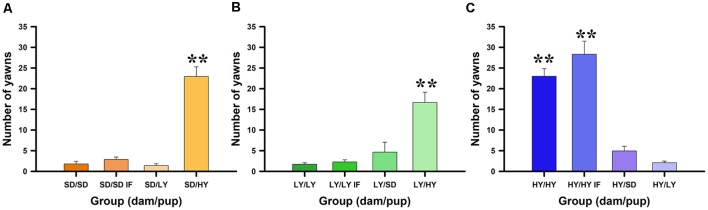
Spontaneous yawning frequency dependents on the dam that take care of male rats. **(A)** Sprague-Dawley (SD) dams do not change the behavioral trend, being the high yawners male rats belonging from HY subline independently of the dam that raised them, see pairs dam/male rats SD/HY (panel **A**), LY/HY (panel **B**), and HY/HY or HY/HY IF (panel **C**); **P < 0.01. Note that LY dams can reduce yawning frequencies in all pairs LY/LY, LY/LY IF or LY/SD (panel **B**). Contrary, HY dams do not increase yawning frequencies in SD or LY male rats (see HY/SD and HY/LY; panel **C**).

The aim of this experiment was to determine which factor has a stronger impact on yawning and penile erection frequencies, the genetic background (sublines or SD male rats), or the raising conditions during the lactation period. Figure 2 shows that spontaneous yawning frequencies did not differ among in-fostered or cross-fostered sublines or SD male rats raised by different dams (see Figures 2A,C). However, HY male rats had a significantly greater number of yawns with respect to their LY and SD counterparts ($\chi_{\left(2\right)}^{2}$ = 44.7, *P* < 0.001). No significant effects were found for the dam subline or SD strain ($\chi_{\left(2\right)}^{2}$ = 3.47, *P* < 0.17) or for the in- or cross-fostering manipulations ($\chi_{\left(1\right)}^{2}$ = 1.91, *P* = 0.17). However, Dunn comparison tests used in the *post hoc* analysis showed a significant difference between HY and LY (*P* < 0.001, Holm–Šidák correction) and between HY and SD (*P* < 0.001) but not between LY and SD male rats (*P* = 0.12; see Figures 2A,C).

**Figure 3:**
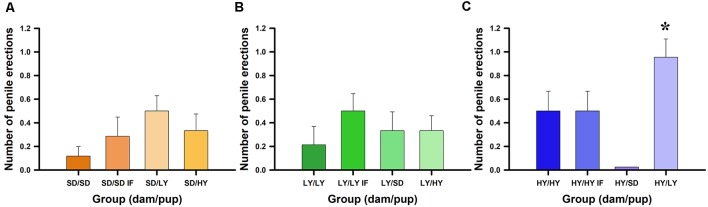
Spontaneous penile erections dependent on the dams that take care of male rats. **(A)** Sprague-Dawley (SD) had similar impact across groups, rearing male rats with lower frequencies of penile erections. **(B)** Low-yawning (LY) dams do not change the penile erection frequencies in the different groups of male rats. **(C)** However, High-yawning (HY) dams are capable to increase the number of spontaneous penile erections on LY male rats (see HY/LY, *P < 0.05).

Figure 3 shows that spontaneous penile erection frequencies significantly differ between HY and LY or SD male rats ($\chi_{\left(2\right)}^{2}$ = 6.90, *P* < 0.05). No significant effects were found with respect to dam subline or SD strain ($\chi_{\left(2\right)}^{2}$ = 0.35, *P* = 0.83) or for in- or cross-fostering manipulations ($\chi_{\left(2\right)}^{2}$ = 0.18, *P* = 0.66; Figures 3A–C). A *post hoc* Dunn test showed significant differences between HY and SD rats (*P* < 0.05, Holm–Šidák correction; Figure 3C), but no significant differences were found between HY and LY (*P* = 0.30) or between LY and SD male rats (*P* = 0.22). Only HY dams were able to significantly increase the number of spontaneous penile erections in male rats (*P* < 0.05, Holm–Šidák correction).

The spontaneous number of grooming bouts showed a significant effect among the different groups of rats tested ($\chi_{\left(2\right)}^{2}$ = 22.40, *P* < 0.001). No significant effects were found for the dams between sublines or with respect to SD dams ($\chi_{\left(2\right)}^{2}$ = 2.17, *P* = 0.33); this was also the case for in- or cross-fostering manipulations ($\chi_{\left(1\right)}^{2}$, *P* = 0.97). A *post hoc* Dunn test showed that HY rats had significantly more grooming bouts with respect to LY and SD rats (*P* < 0.001). No significant differences were found among the male rats tested (*P* = 0.08, data not shown).

### Experiment 2. Maternal Care Organization in the Cross-fostered Male Rats Raised by HY, LY, or SD Dams

**Figure 4:**
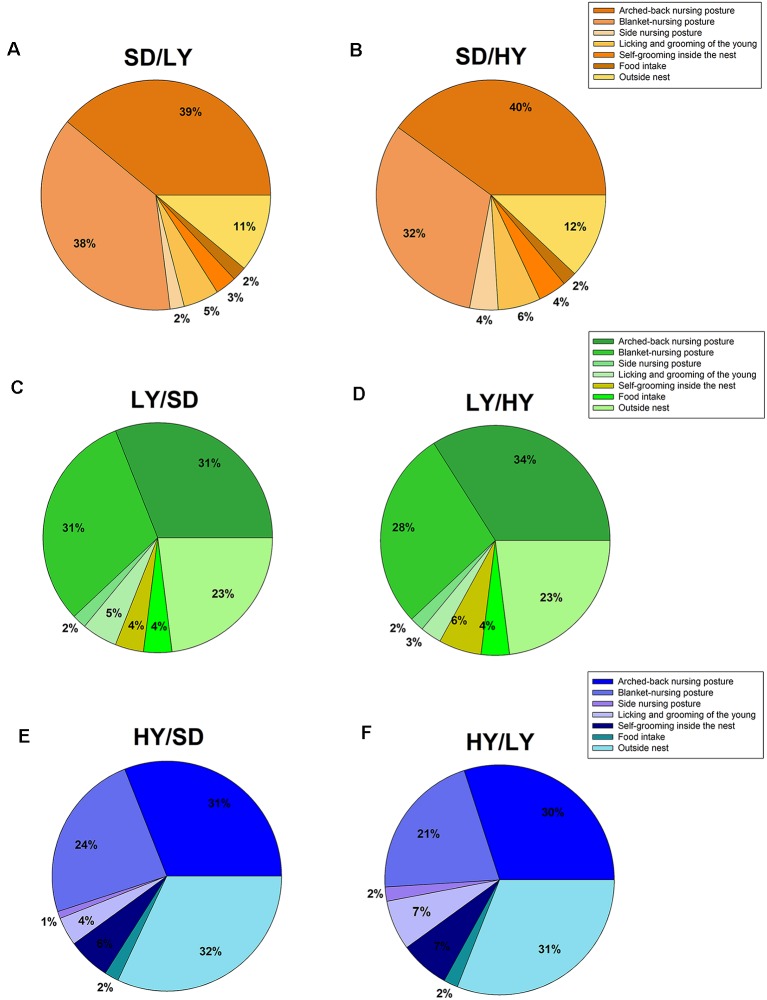
Maternal care in cross-fostered male rats raised by HY or LY sublines or by SD dams. **(A)** SD dams that raised LY male rats (SD/LY) did not differ in their maternal behavior components. **(B)** SD dams showed an increase time spend outside the nest, but the difference was not significant (SD/HY). **(C,D)** LY dams showed a decreased frequency of the arched-back nursing posture compared to SD dams (SD/LY) or with respect to LY dams that raised HY male rats (LY/HY). **(E,F)** HY dams showed a lower proportion of blanket-nursing posture with respect to SD (HY/SD) or LY rats (HY/LY).

The aim of this experiment was to determine the changes in the behavioral components of maternal care due to cross-fostering among the sublines and SD rats. A beta family GLM was used to analyze the proportion of time for each of the maternal components displayed in the early lactation period and showed that arched-back and blanket-nursing postures differed among the sublines and SD rats ($\chi_{\left(2\right)}^{2}$ = 22.33, *P* < 0.001 and $\chi_{\left(2\right)}^{2}$ = 26.78, *P* < 0.001, respectively) as illustrated in Figure 4, which shows the percentage of time SD dams (Figures 4A,B, orange pie charts) spent in each maternal behavioral component on cross-fostered male rats from both sublines. Figure 4 shows the maternal behavioral components displayed by LY (Figures 4C,D, green pie charts) and HY (Figures 4E,F, blue pie charts) dams. The dams differed in the time spent in self-grooming in the nest ($\chi_{\left(2\right)}^{2}$ = 14.31, *P* < 0.001), as well as in the time spent outside the nest ($\chi_{\left(2\right)}^{2}$ = 45.90, *P* < 0.001), but not in the other maternal behaviors measured. In general, both sublines showed a higher proportion of blanket-nursing posture and time spent outside the nest area with respect to that displayed by SD dams (*P* < 0.05). The latency to approach the litter after cross-fostering is higher in HY dams than in SD dams (*P* < 0.05).

**Figure 5:**
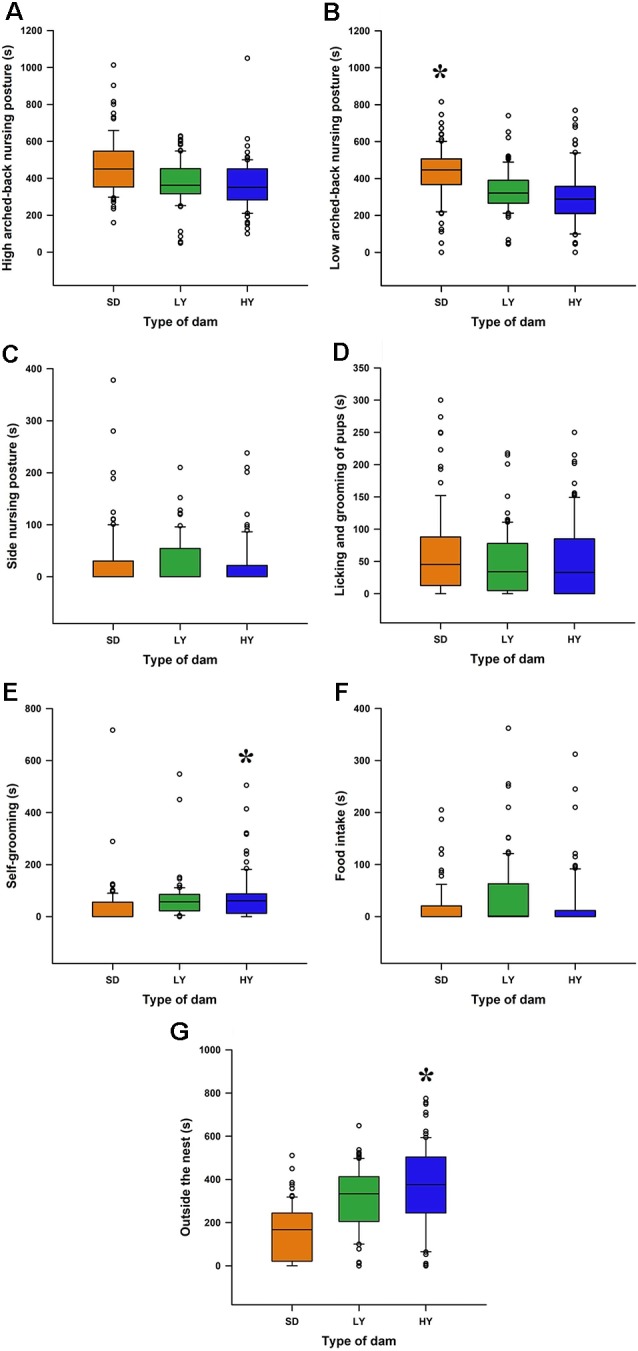
Spontaneous maternal care display in the early lactation period in HY and LY dams or in SD dams. **(A)** High arched-back nursing posture did not differ between sublines or with respect to SD dams. **(B)** SD dams had a higher time spent in the low arched-back nursing position with respect to HY and LY dams (*P < 0.05). **(C)** The time spent in the side-nursing posture was similar between the sublines or in SD dams. **(D)** The time spent in licking or grooming the pups was similar among HY, LY, or SD dams. **(E)** The time spent in self-grooming is higher in HY dams with respect to LY or SD dams (*P < 0.05). **(F)** The time the dams spent on eating pellets was similar in the different group of rats. **(G)** HY dams spent more time inside the nest with respect to LY or SD dams (*P < 0.05).

Note that HY, LY, and SD dams showed similar values to those obtained in a previous study, but in this study, the maternal components were determined without disturbing the mother and their pups; that is, we measured the ongoing maternal behavior without disturbing them (Figure 5). Note that HY and LY dams showed fewer arched-back nursing postures than SD dams (*P* < 0.05). Dams from the HY subline spent more time in self-grooming (*P* < 0.05; Figure 5E) and outside the nest (*P* < 0.05; Figure 5G) than LY and SD dams.

### Experiment 3. Systemic Administration of (−)-Quinpirole Differentially Increased Yawning and Penile Erection and Decreased Grooming and Scratching Frequencies in Both Sublines and SD Rats

**Figure 6:**
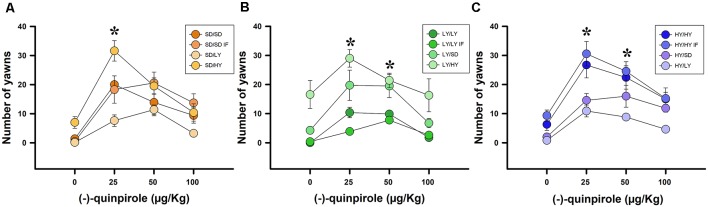
Systemic administration of (−)-quinpirole increased yawning frequencies, which was dependent on the phenotype of male rats. **(A)** SD dams that raised HY male rats yawned significant higher with 25 mg/kg of quinpirole with respect to the rest of pairs (SD/HY, *P < 0.05). **(B)** LY male rats had the lowest yawning frequencies when raised by a dam of their own phenotype, with the highest frequency of yawning occurring when LY dams raised HY male rats (LY/HY; *P < 0.05). **(C)** HY male rats have the greatest yawning frequencies independent of the phenotype of the dams that raised them. Note that HY/HY IF and HY/HY had significantly higher yawning frequencies with respect to HY/SD and HY/LY (*P < 0.05).

The aim of this experiment was to analyze whether the cross-fostering manipulation changed the sensitivity to the D_2_ dopaminergic agonist (−)-quinpirole. A beta family GLM was fitted using the yawning frequency as the dependent variable. Systemic injections of different doses of (−)-quinpirole had a significant effect on yawning frequencies ($\chi_{\left(2\right)}^{2}$ = 9.66, *P* < 0.01; Figure 6). There was also evidence for a significant effect of the subline or SD strain of the male rats ($\chi_{\left(2\right)}^{2}$ = 96.20, *P* < 0.001), with the HY rats being significantly more responsive to this agonist than LY or SD rats. At larger dosages, the yawning frequency decreased, which significantly contributed to the linear regression model ($\chi_{\left(2\right)}^{2}$ = 165.99, *P* < 0.001). Finally, a significant interaction was found between the sublines or SD male rats and the (−)-quinpirole dose ($\chi_{\left(2\right)}^{2}$ = 17.62, *P* < 0.001; Figure 6). The estimated ED_50_ was 4.27 μg/kg for HY rats; in the case of SD rats, the estimated ED_50_ for male rats reached 5.96 μg/kg, which is 1.39 times higher than that of the HY subline, and the estimated ED_50_ for LY rats reached 7.86 μg/kg, which is 1.84 times higher than that of the HY subline. We did not find evidence against the null hypothesis for the dam subline, either for in- or cross-fostered manipulations or in the latency to approach their litter ($\chi_{\left(2\right)}^{2}$ = <1, *P* > 0.05).

**Figure 7:**
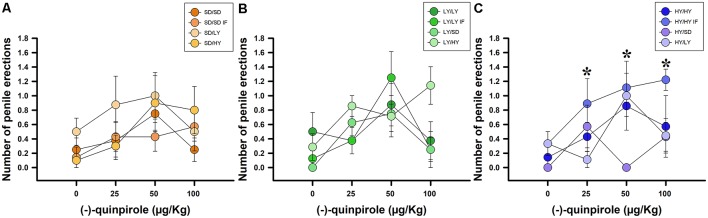
Systemic administration of (−)-quinpirole increased penile erection frequencies in all groups of rats. **(A)** SD dams that raised HY male rats (SD/HY) showed higher penile erection frequencies with respect to the other pairs of dams/male rats. **(B)** LY male rats do not differ in penile erection frequencies with respect to the phenotype of the dam that raised them. **(C)** HY male rats in-fostered with another HY dam (HY/HY IF) had the highest penile erection frequencies with respect to the other pairs of HY male rats (*P < 0.05).

A binomial family GLM was fitted using the occurrence of penile erections as the dependent variable. Systemic administration of (−)-quinpirole had a significant effect on the log-odds of HY penile erections ($\chi_{\left(2\right)}^{2}$ = 10.53, *P* < 0.01; Figure 7). Additionally, there was evidence for an effect of the different groups of rats on the logarithm of the probability of penile erections ($\chi_{\left(2\right)}^{2}$ = 14.51, *P* < 0.001), as well as an interaction between the type of rats and the dosage of (−)-quinpirole ($\chi_{\left(2\right)}^{2}$ = 11.823, *P* < 0.01; Figure 7). The estimated ED_50_ for HY rats was 32.23 μg/kg, and for SD rats, the estimated dose was 144.2 μg/kg, which is 4.47 higher than that for HY male rats. In the case of the LY subline, the ED_50_ could not be computed because the drug decreased the odds of penile erections in the case of this subline instead of increasing them. We did not find evidence against the null hypothesis for the dams, either in- or cross-fostered rats or for the latency to approach the litter by the dams ($\chi_{\left(2\right)}^{2}$ = <1, *P* > 0.05).

A Poisson family GLM was fitted using the frequency of grooming episodes as the dependent variable. The dose of (−)-quinpirole was found to have a significant inhibitory effect on grooming bouts ($\chi_{\left(2\right)}^{2}$ = 159.78, *P* < 0.001). A significant effect was also found for the group of rats ($\chi_{\left(2\right)}^{2}$ = 63.29, *P* < 0.001) and the interaction of the group of rats and dosage ($\chi_{\left(2\right)}^{2}$ = 54.42, *P* < 0.001).

The ED_50_ was estimated as the middle point between grooming bouts at baseline and total inhibition, with zero mean grooming bouts expected (see Supplementary Table S1). Thus, the estimated ED_50_ for HY rats was 42.76 μg/kg, for SD rats was 29.72 μg/kg, and for LY rats was 140.90 μg/kg, which was 3.29 times higher than that of HY rats. We did not find a significant effect of the dams that took care of the different male rats or for in- or cross-fostered manipulations ($\chi_{\left(2\right)}^{2}$ = <1, *P* > 0.05, Supplementary Table S1).

A binomial family GLM (logistic regression) was fitted using the occurrence of scratching as the dependent variable. There was a significant inhibitory effect of (−)-quinpirole on the odds of scratching ($\chi_{\left(2\right)}^{2}$ = 37.019, *P* < 0.001). We did not find an effect of the pup’s subline or SD, the dam’s subline or SD, or the in-fostering or cross-fostering manipulation. As no subline effects were detected, a single ED_50_ was estimated at 53.31 μg/kg (Supplementary Table S2).

### Experiment 4. Dopamine D_2_ Receptor Expression in the Striatum

The aim of this experiment was to determine the D_2_ receptor relative expression in the striata of HY, LY, and SD of male rats. Two main immunoreactive bands were observed around 50 kDa. We measured these both as coincided with the technical specifications for the antibody indicating additional bands could be observed in a range from 60 to 100 kDa.

**Figure 8:**
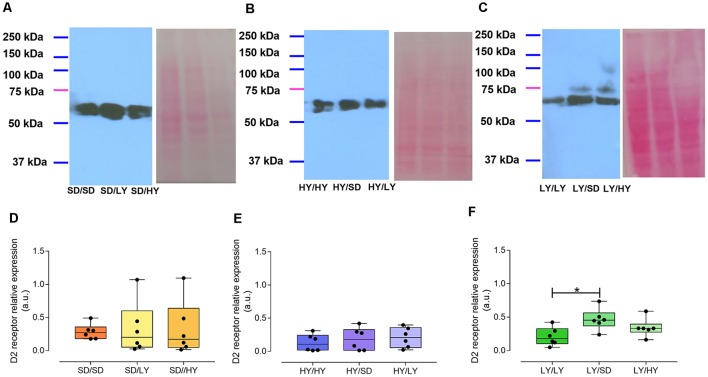
Relative expression of the D_2_ receptor (DR2) in the striatum of rats of both sublines and of SD male rats raised by different dams. Representative immunoblots from the striatal D_2_ receptor expression and matched Ponceau’s red staining in **(A)** SD male rats raised by SD dams (SD/SD), or by LY (SD/LY), or HY (SD/HY) male rats; **(B)** HY, SD, or LY male rats raised by HY dams (HY/HY, HY/LY, and HY/SD); **(C)** LY, SD, or HY male rats raised by LY dams (LY/LY, LY/SD, and LY/HY, respectively). The D_2_ receptor relative expression was normalized against the matching Ponceau’s red stained lane and expressed as arbitrary units. **(D–F)** Data are shown as the median and interquartile ranges. Only SD male rats raised by LY dams significantly differ from LY/LY group [Kruskal–Wallis analysis of variance (ANOVA), *H* = 7.4, *P* < 0.02, followed by Dunn multiple-comparisons test, *P < 0.02].

The levels of the striatal D_2_ receptor expression in both sublines or in SD male rats did not significantly differ in the different groups of rats raised by SD dams (Figures 8A,D). Furthermore, HY dams did not affect the expression of D_2_ dopamine receptors in SD, LY, or HY male rats (Figures 8B,E). However, SD male rats reared by LY dams showed a significant increase in the striatal dopamine D_2_ receptor expression but not in HY male rats (Figures 8C,F, respectively; Kruskal–Wallis ANOVA, *H* = 7.4, *P* < 0.02; followed by Dunn test, *P* < 0.02).

## Discussion

Our results showed that the higher number of spontaneous and (−)-quinpirole-induced yawning bouts and penile erections in HY male rats is independent of the type of dam that raises them, supporting that the yawning frequency is specific to each subline. Therefore, genetic backgrounds are the main component due to strong inbreeding, and nurturing conditions during the lactation period did not play a significant role in determining yawning frequencies (Figure 2). A similar trend was obtained with LY and SD male rats, which had lower yawning frequencies independent of the type of dam that raised them, supporting that the genetic background is the main variable that determines yawning frequency.

In the case of the spontaneous number of penile erections, we clearly demonstrated that HY dams were able to significantly increase the number of penile erections in LY male rats. It is relevant that male rats subjected to in-fostering generally had almost double the number of penile erections with respect to their nonfostered counterparts (Figure 3), but these differences did not reach significant levels due to, at least in part, the higher variability and lower incidence of noninduced penile erections. It has been demonstrated that the amount of prepubescent self–anogenital grooming is greater in male rats than in their female counterparts, and this grooming component is androgen-dependent because castration reduced it in male rats and androgenization in female rats increased it (Moore, 1986a). The amount of anogenital grooming is also context dependent because living in isolation conditions significantly increased genital grooming (Moore, 1986b). In our conditions, all groups of rats lived in groups, with 3–4 Ss in acrylic cages, and tests were performed in transparent glass cylinders. Therefore, considering that all the conditions are equal, it was outstanding and unexpected that HY dams could increase the number of penile erections in the LY male rats (Figure 3C), supporting a strong maternal component in the expression of penile erections out of a sexual context.

It has been demonstrated that cross-fostering is a postnatal environmental manipulation that is capable of inducing behavioral and physiological changes in mice and rats (Bartolomucci et al., 2004). In fact, mother–pup interaction in rats occurs within the nest and consists of approaching the litter, licking and grooming the pups, and nursing them in different positions (Fleming et al., 1999). To control the effects of manipulation due to the fostering of the pups, we also used an in-fostering group of rats as an additional control of pup manipulation, but we obtained an increase in yawning frequencies and in penile erections in in-fostered male rats, supporting that dams are able to discern that they are different pups and change in some way the strategy in which they care for them, which is not reflected in the percentage of time spent in the different behavioral measurements. However, these changes are due to neonatal handling, as already demonstrated in the Roman lines (Fernández-Teruel et al., 1997). It is important to mention that Roman lines share various behavioral characteristics with our yawning sublines (Melis et al., 2019).

Our results also showed that arched-back and blanket-nursing postures differed between both sublines, as well as the time spent in self-grooming and outside the nest or even the time that HY dams approach the litter, supporting a different organization of maternal care in the HY subline with respect to the LY and SD dams, as previously demonstrated (Ugarte et al., 2011). Importantly, the general trend is maintained within each group of dams instead of the characteristic organization of maternal care in the different fostered groups evaluated in these experiments as HY dams that had a different organization of maternal care (Ugarte et al., 2011). These results clearly support that the genetic background between the sublines is quite stable because, as already mentioned, HY and LY rats were maintained by a strict inbreeding process for more than 85 generations (~35 years), which implies that the presence of different pups with different genetic pools is not able to change maternal care. A similar trend has been obtained with the naturally occurring variations in maternal licking/grooming of pups that were significantly correlated when the mother nursed in the arched-back position and persisted in the first and even second litters, supporting a possible epigenetic mechanism (Caldji et al., 2000). In future experiments, we could analyze the relationship of the amount of anogenital grooming displayed by the mother and the frequency of spontaneous and drug-induced penile erections in both sublines in the first and second generations to evaluate persistent effects due to maternal care.

In this context, it is relevant that Fleming et al. (1999) showed that there is an interaction between the newborn and the mother that is capable of altering the basic mechanisms of behavioral expression in both, including in neuroanatomical, neurochemical, and behavioral aspects (Fleming et al., 1999). If we consider the degree of interaction of the dams with their offspring and the ways that individuals respond later in life and then changing the response of hypothalamic–hypophysis–adrenal gland axis regulation and affiliative and social behaviors (Fleming et al., 1999), in which yawning and grooming play a central role (Collins and Eguibar, 2010; Melis et al., 2019). So, nurture had a strong influence on behavior, but it is not the case with yawning and penile erections in our experimental conditions. Because yawning is an emphatic behavior that is also related to communication, it is highly remarkable that maternal care did not influence the spontaneous expression of yawning, and in the case of HY male rats, they were noted as the rats that yawned more with respect to LY and SD rats independent of the subline or SD dams that raised them. Another possibility is that social interaction between the dams and their littermates can reverse the effects due to the quality and quantity of maternal care.

A similar trend was obtained with spontaneous penile erections, with HY rats showing more of this spinal reflex without any sexual context, neither motivational nor somesthetic. In future experiments, we will analyze the penile erections induced by an inaccessible estrous female, the so-called noncontact penile erections, or in the case of the retraction of the preputial skin as a mechanism to increase the frequency of penile erections in both sublines (Sachs et al., 1988; Sachs, 2000). As we already demonstrated in sexually experienced male HY rats, they showed a different copulatory pattern because they had more sexual bouts, with longer interintromission intervals that delayed ejaculation (Eguibar et al., 2016). It is quite relevant that HY dams are able to significantly increase the number of penile erections in the LY male rats, and this effect is probable due to an increase in the genital grooming given by HY dams because the number of penile erections in adulthood is strongly dependent on the genital grooming displayed in the first week of life (Moore, 1986a, b). This is the first report that shows an increase in the number of penile erections after a cross-fostering technique, which implies that early-life experiences have an important impact on the central and/or peripheral mechanisms that participate in penile erections. Another possible explanation is that maternal care is capable of changing oxytocinergic and dopaminergic neurotransmission and perhaps nitric oxide levels in key integrative areas in the hypothalamus, such as in the PVN of the hypothalamus, which are capable of increasing penile erections (Argiolas, 1994; Giuliano and Rampin, 2000). In fact, the local administration of oxytocin, dopaminergic agonist, or even nitric oxide increased the number of penile erections, supporting a central role of the PVN (Argiolas, 1994).

In a previous study, we showed that HY male rats have a higher percentage of noncopulators with respect to SD rats (Portillo et al., 2010). In Roman high-avoidance and in the novel exploration higher responder sublines, all of them showed deficits in sexual behavior that are proposed to be due to changes in the dopaminergic tone (Melis et al., 2019). Another group of rats selected on the basis of susceptibility to cholinergic agonists was the sensible and resistant Flinders sublines, FSL and FSR, respectively. These two groups of rats also differed in their sexual performance because both had a marked decrease in the ejaculatory frequencies and reached exhaustion sooner with respect to SD controls (Ferreira-Nuño et al., 2002). Importantly, the FSL rats had higher levels of anxiety and depressive-like behavior that were like those obtained in the LY subline of rats (Overstreet, 1993; Overstreet et al., 2005; Eguibar et al., 2017b). When cross-fostered with SD dams, FSL rats did not show a change in depressive-like symptoms in the forced swing test, and in adulthood, an SD liter showed clearly deteriorated depressive-like behavior when raised by FSL dams (Malkesman et al., 2008). Similar results were obtained when cross-fostering was used between stress-vulnerable and stress-resilient rats (Uchida et al., 2010).

These results are relevant, considering that HY male rats being less emotionally reactive (Moyaho et al., 1995) and having an increased frequency of grooming bouts when exposed to a novel environment (Eguibar and Moyaho, 1997), supporting this hypothesis. A recent experiment showed that HY rats explore and enter more the open arms of an elevated plus maze, supporting that they are resilient to stress manipulations (Eguibar et al., unpublished results). Cross-fostering between Lewis rats, a group of rats with higher anxiety rates, and Fischer 344 rats, which are resilient to stress, is relevant. When cross-fostering was performed, dams induced a nonsignificant increase in the offspring’s time spent in the open arms in the elevated plus-maze, but they were able to induce an increase in the offspring’s exploration of the center in an open-field arena, supporting that maternal care was able to change the anxiety responses (McCarty, 2017; Siviy et al., 2017).

Another relevant aspect is that maternal care can impact spontaneous penile erections, but this is not the case when we determined D_2_-like dopaminergic agonist-induced yawning and penile erections, in which the HY phenotype had higher frequencies independent of the dams that raised them. It was unexpected that LY dams were capable of increasing dopamine D_2_ receptor expression in the striatum in SD male rats, supporting that, during neurodevelopment, maternal care and the presence of their siblings and nest environment are capable of changing dopaminergic function independent of the genetic background of the male rats, but not in the (−)-quinpirole-induced yawning and penile erections. We use a cumulative dosing because it allows us to quickly evaluate behavioral responses to this dopaminergic agonist in a valuable group of rats as they are the in- and cross-fostered which require shorter time to obtain a dose–response curve with similar distribution to that obtained with other dose schemes. Previously this type of approach has been used in rats under food restriction that decreases or with high-fat diet that enhances sensitivity to the actions of quinpirole (Baladi and France, 2009). It is also the case with streptozotocin-treated rats that had a reduced response to quinpirole-induced yawning (Sevak et al., 2007).

Additionally, (−)-quinpirole decreased grooming bouts and abolished scratching; in the case of grooming, this effect was mainly due to the stimulation of D_1_ receptors (Berridge and Aldridge, 2000a, b; Eguibar et al., 2003). Our results showed a clear inhibitory effect in all groups of rats, with the HY rats being more sensitive than the LY and SD rats, supporting the participation of D_2_-like dopamine receptors in the modulation of this behavior. Dopamine had a modulatory role in the striatum because it is a key component for the serial order of the sequence of grooming components, including the transitions in the face components through the lateral parts of the body (Aldridge and Berridge, 1998). It is relevant that in mice with a knockdown mutation in the dopamine transporter gene, extracellular dopamine levels in the striatum increase by 170% with respect to wild-type counterparts, and they show an increased grooming stereotypy that emulates obsessive–compulsive disorder (Berridge et al., 2005). In animal models of obsessive–compulsive symptoms, there were excessive dopamine levels, and it is relevant that subchronic treatment of (−)-quinpirole produced enhanced checking behavior in the open-field arena (Szechtman et al., 1998). Our results of decreased grooming support a role for dopamine D_2_ receptors in this behavior, which can be a display of obsessive–compulsive disorder, as previously reported (Robbins et al., 2019), but further experimental data are necessary to support the role of each type of dopamine D_2_-like receptor. HY male rats had higher D_1_ binding in the striatum with respect to LY rats, which supports their higher grooming bout frequency (Eguibar and Moyaho, 1997; Díaz-Romero et al., 2005).

Scratching behavior depends directly on a spinal command and can be modulated by higher brain centers that are capable of exciting or inhibiting the activity of the scratch generator (Gelfand et al., 1988). On the other hand, central administration of gastric releasing hormones and related peptides, such as bombesin, induced scratching, and this spinal reflex can be inhibited by the administration of D_1_ antagonists, such as SCH 23390 (Van Wimersma Greidanus and Maigret, 1991), supporting again a role of dopamine neurotransmission in scratching. In HY rats, intracerebroventricular administration of bombesin significantly reduced yawning frequency and increased scratching independent of grooming bouts (Díaz-Romero et al., 2002).

## Conclusion

Our results showed that yawning is a stereotyped and genetically determined behavior with little influence of initial stages of development, including maternal care and nesting conditions and sibling interactions during the lactation period. In fact, the behavioral responses after the administration of dopaminergic D_2_-like agonist (−)-quinpirole clearly showed higher responses in the HY male rats, supporting a strong role of the genetic background.

In future experiments, we will analyze the specific role of dopaminergic neurotransmission in the PVN of the hypothalamus or in the striatum by applying dopaminergic agonists directly to each area. The increase in spontaneous penile erections in LY male rats raised by HY dams and the increase in D_2_ receptor expression levels induced by LY dams In SD male rats require further experimental data, but these results support a specific role of maternal care in the expression of yawning and penile erections changing some biochemical characteristics in the central nervous system taking into account due to the advantage of high spontaneous expression of yawning and penile erections of HY subline with respect to SD and even Wistar rats.

## Data Availability Statement

All datasets generated for this study are included in the article and are available by request.

## Ethics Statement

The animal study was reviewed and approved by Benemérita Universidad Autónoma de Puebla Animal Care and Use Committee No. EGC SAL-G-2019.

## Author Contributions

Tasks of individual authors: JE and CC: conceptualization. ÁD-N, ÁC, CC, AU, AT, and JE: data curation and investigation. ÁD-N, ÁC, HC-F, and JE: formal analysis. CC and JE: funding acquisitions and resources. ÁD-N, CC, ÁC, and JE: methodology. ÁD-N, CC, AT, and JE: writing.

## Conflict of Interest

The authors declare that the research was conducted in the absence of any commercial or financial relationship that could be construed as a potential conflict of interest.
