# Supplementary material for: Yawning and Penile Erection Frequencies Are Resilient to Maternal Care Manipulation in the High-Yawning Subline of Sprague–Dawley Rats
Source: Front Behav Neurosci. 2020 Mar 12;14:20. doi: 10.3389/fnbeh.2020.00020 (PMC7080979; doi:10.3389/fnbeh.2020.00020)
Supplement: Supplementary file 1 [file Table_1.DOCX]

Table 1. Grooming bouts induced by subcutaneous injection of (-)-quinpirole at different doses in the in-fostering and cross-fostering male rats

| **Group (Dam/pup)** | **(-)-Quinpirole dose (µg/Kg)** | | | |
| --- | --- | --- | --- | --- |
|  | **0** | **25** | **50** | **100** |
| **SD/SD** | 5.9 ± 0.8 | 2.4 ± 0.6 | 0.9 ± 0.5** | 0.9 ± 0.3** |
| **SD/SD IF** | 4.6 ± 0.9 | 1.7 ± 0.8 | 1.4 ± 0.5* | 0.4 ± 0.2** |
| **SD/LY** | 5.1 ± 1.0 | 1.7 ± 0.9 | 1.5 ± 1.6 | 2.5 ± 1.0 |
| **SD/HY** | 3.1 ± 0.4 | 0.4 ± 0.2** | 0.6 ± 0.3** | 0.9 ± 0.4** |
| **LY/LY** | 3.4 ± 0.5 | 2.2 ± 0.6 | 3.0 ± 0.5 | 2.7 ± 1.2 |
| **LY/LY IF** | 4.2 ± 1.3 | 1.5 ± 0.7 | 2.4 ± 1.4 | 1.7 ± 0.5 |
| **LY/SD** | 9.5 ± 1.6 | 1.6 ± 0.7* | 1.1 ± 0.5* | 0.7 ± 0.4*** |
| **LY/HY** | 2.3 ± 0.3 | 1.0 ± 0.4* | 0.1 ± 0.1*** | 1.3 ± 0.8* |
| **HY/HY** | 3.7 ± 0.3 | 1.1 ± 0.4* | 0.7 ± 0.4** | 1.1 ± 0.4* |
| **HY/HY IF** | 3.8 ± 0.6 | 1.0 ± 0.5 | 0.6 ± 0.2** | 0.6 ± 0.2** |
| **HY/SD** | 7.0 ± 0.9 | 2.4 ± 0.8 | 1.4 ± 0.5* | 1.8 ± 0.8* |
| **HY/LY** | 3.8 ± 0.5 | 1.3 ± 1.5 | 2.2 ± 0.7 | 2.3 ± 0.5 |

The data are the mean ± E.E.M. SD=Sprague-Dawley; HY=high-yawning; LY=low-yawning. IF=In-fostering technique. * *P<* 0.05, ** *P<* 0.01; *** *P<* 0.001.
