# Supplementary material for: Yawning and Penile Erection Frequencies Are Resilient to Maternal Care Manipulation in the High-Yawning Subline of Sprague–Dawley Rats
Source: Front Behav Neurosci. 2020 Mar 12;14:20. doi: 10.3389/fnbeh.2020.00020 (PMC7080979; doi:10.3389/fnbeh.2020.00020)
Supplement: Supplementary file 2 [file Table_2.DOCX]

Table 2. Scratching induced by different doses of (-)-quinpirole in the different groups of in-fostering and cross-fostering male rats

| **Group (Dam/pup)** | **(-)-Quinpirole dose (µg/Kg)** | | | |
| --- | --- | --- | --- | --- |
|  | **0** | **25** | **50** | **100** |
| SD/SD | 3.6 ± 2.1 | 1.6 ± 0.9 | 0.5 ± 0.3** | 0.1 ± 0.1*** |
| SD/SD IF | 2.6 ± 1.4 | 1.7 ± 1.7 | 0.3 ± 0.2** | 0.0 ± 0.0*** |
| LY/SD | 3.2 ± 1.0 | 0.6 ± 0.4** | 0.1 ± 0.1*** | 0.4 ± 0.2** |
| HY/SD | 2.0 ± 1.5 | 0.6 ± 0.4 | 0.0 ± 0.0*** | 0.1 ± 0.1*** |
| LY/LY | 1.9 ± 0.7 | 0.5 ± 0.3 | 0.1 ± 0.1*** | 0.6 ± 0.5* |
| LY/LY IF | 0.0 ± 0.0 | 0.6 ± 0.3 | 0.0 ± 0.0*** | 0.0 ± 0.0*** |
| SD/LY | 0.9 ± 0.4 | 0.2 ± 0.2 | 0.1 ± 0.1* | 0.1 ± 0.1* |
| HY/LY | 2.3 ± 0.9 | 0.4 ± 0.1** | 0.5 ± 0.2** | 0.3 ± 0.2** |
| HY/HY | 1.7 ± 0.5 | 0.1 ± 0.1** | 0.0 ± 0.0*** | 0.3 ± 0.2** |
| HY/HY IF | 3.3 ± 1.3 | 0.1 ± 0.1** | 0.4 ± 0.2* | 0.1 ± 0.1** |
| SD/HY | 1.2 ± 0.9 | 0.1 ± 0.1* | 0.0 ± 0.0*** | 1.0 ± 1.0 |
| LY/HY | 3.2 ± 1.3 | 0.4 ± 0.2* | 0.7 ± 0.4* | 0.3 ± 0.2** |

The data are the mean ± E.E.M. SD=Sprague-Dawley; HY=high-yawning; LY=low-yawning. IF=In-fostering technique. * *P<* 0.05, ** *P<* 0.01; *** *P<* 0.001.
